# Cæarean Operation

**Published:** 1855-11

**Authors:** 


Cæsarean Operation.—Professor Stoltz, of Strasbourg, after relating a case that has occurred to himself, in which this operation has been performed twice upon the same subject with success, refers to no less than seventeen similar cases which have been authentically recorded during the present century. He protests against the rule, now absolute in Paris, on account of the invariable mortality of this operation there, of preferring induction of premature labor to the Cæsarean section, being made applicable to the country at large.—(*Gaz. Méd.* 1855. No. 25.) *Ibid.*

